# Prevalence and determinants of pre-hypertension and hypertension among the adults in rural Bangladesh: findings from a community-based study

**DOI:** 10.1186/s12889-015-1520-0

**Published:** 2015-02-28

**Authors:** Masuma Akter Khanam, Wietze Lindeboom, Abdur Razzaque, Louis Niessen, Abul Hasnat Milton

**Affiliations:** Centre for Control of Chronic Diseases in Bangladesh, icddr,b, Dhaka, 1212 Bangladesh; Centre for Clinical Epidemiology and Biostatistics (CCEB), School of Medicine and Public Health, the University of Newcastle, Callaghan, NSW 2308 Australia; Cardialysis in Rotterdam, Rotterdam, the Netherlands; International Centre for Diarrhoeal Diseases Research, Bangladesh (icddr,b), Dhaka, Bangladesh; Professor of Health Economics, Liverpool School of Tropical Medicine, Pembroke Place, Liverpool, L3 5QA UK; International Health, Johns Hopkins Bloomberg School of Public Health, 615 N Wolfe Street, Baltimore, MD 21205 USA

**Keywords:** Hypertension, Prehypertension, Bangladesh, Cross sectional study

## Abstract

**Background:**

The people of low and middle income countries bear about 80% of the global burden of diseases that are attributable to high blood pressure. Hypertensive people contribute half of this burden; the rest is among the people with lesser degrees of high blood pressure. Prehypertension elevates the risk of CVD, and that of end-stage renal disease. Bangladesh is a developing country, with more than 75% of the population live in rural area. This study aims to determine the prevalence and predictors of pre-hypertension and hypertension among the adults in rural Bangladesh.

**Methods:**

A cross-sectional study of major non-communicable disease risk factors (tobacco and alcohol use, fruit and vegetable intake, physical activity) was conducted in rural surveillance sites of Bangladesh. In addition to the self-reported information on risk factors, height and weight, and blood pressure were measured during household visits using standard protocols of the WHO STEPwise approach to Surveillance. The study population included 6,094 men and women aged 25 years and above. Adjusted and unadjusted logistic regression analyses were performed to evaluate the association of prehypertension and hypertension with various factors.

**Results:**

The prevalence of pre-hypertension and hypertension was 31.9% and 16.0%, respectively. The men had a higher prevalence (33.6%) of pre-hypertension compared to the women (30.3%). Multivariate analysis showed that increasing age [OR 2.30 (1.84-2.87)] and higher BMI [OR 4.67 (3.35-6.51) were positively associated with pre-hypertension. For hypertension, multivariate analysis showed that increasing age [OR 4.48 (3.38-5.94)] and higher BMI (specially the overweight category) was positively associated.

Significant linear relationships of prehypertension were found with age [P for trend < 0.0001] and BMI [P for trend < 0.0001]. Linear regression for hypertension shows significant association with age [P for trend < 0.0001] but not with BMI [P for trend 0.3783].

**Conclusion:**

Approximately one third and one-sixth of the adult population of rural Bangladesh are affected with pre-hypertension and hypertension, respectively. This poses a great challenge ahead, as most of the people with pre-hypertension will progress towards hypertension until otherwise undergo in any pharmacological or lifestyle intervention.

## Background

High blood pressure exerts a major share in the global burden of disease, and it is unduly higher in the low income countries than in the high income countries [1]. Hypertensive people contributes half of this burden; the rest was among the people with lesser degrees of high blood pressure [2]. More specifically, elevated blood pressure is responsible for approximately 60% of stroke and over 50% of ischemic heart disease [1]. Prehypertension elevates the risk of CVD [3], and that of end-stage renal disease [4]. Prehypertension is associated with CVD mortality, especially stroke mortality [3] and stroke morbidity [5]. Pre-hypertension is now recognized as a potential candidate for cardiovascular intervention or risk reduction.

There is no definite lower threshold of blood pressure for potential danger of cardiovascular mortality [6,7]. The people with high normal blood pressure (systolic blood pressures (SBP) from 120 to 139 mmHg and/or diastolic blood pressures (DBP) from 80 and 89 mmHg) develop hypertension faster and in an increased risk of cardiovascular disease [8,9]. The Seventh Report of the Joint National Committee on Prevention, Detection, Evaluation, and Treatment of High Blood Pressure (JNC 7), introduced the new category of “pre-hypertension”, defined as systolic BP of 120 to 139 mm Hg and/or diastolic BP of 80 to 89 mm Hg [10]. Recent studies found an association between pre-hypertension and increased risk of Coronary Artery Disease [11,12]. Follow up studies also reported that prehypertension is an independent risk factor for cardiovascular and cerebrovascular diseases [13]. A recent meta-analysis reports that even lower range of prehypertension is associated with higher risk of cardiovascular disease (CVD) [14]. Prehypertension is also associated with chronic kidney diseases [15-17]. In USA, over 9% of death and approximately 3.5% of hospitalizations are attributable to pre-hypertension [18].

In USA, the prevalence of prehypertension ranged between 31% [19] to 48.2% [20]. In the neighbor country, India, the prevalence stretched from 32% to 44% [21,22]. The prevalence is 21.9% in Chinese population [23].

Evidence now exists that prehypertension leads to the development of frank hypertension [9,24], and that the development of full blown hypertension can be prevented with administering antihypertensive medications to the patients with prehypertension [25,26]. On the basis of the 7th JNC, pre-hypertension necessitates daily life adjustments to prevent development to hypertension [10].

Bangladesh is a low income, developing country, where more than 75% of the population lives in rural area. There is no data reporting the prevalence of pre-hypertension and the factors associated to it in Bangladesh. In this study we aim to find the prevalence and identify predictors of pre-hypertension among the adults in rural Bangladesh. Additionally, we determined the prevalence and predictors of hypertension among the study population. We also compared the determinants for pre-hypertension and hypertension.

## Methods

### Ethical considerations

The study was conducted according to the principles expressed in the Declaration of Helsinki. The protocol for the Non Communicable Disease (NCD) risk factor survey using the World Health Organization STEPwise approach to Surveillance (WHO STEPS) [27] was approved by the Scientific Board of the International Network for the Demographic Evaluation of Populations and Their Health in Developing Countries (INDEPTH Network) and was also in accordance with the ethics codes of surveillance sites of International Centre for Diarrhoeal Diseases Research, Bangladesh (ICDDR,B). Ethical approval was also obtained from the Human Research Ethics Committee, The University of Newcastle, Australia.

Both verbal and written informed consent was obtained from the study participants, and they had the right to withdraw themselves from the study at any time.

#### Setting and sample size

The study population was obtained from three rural demographic surveillance sites, Matlab, Abhaynagar and Mirsarai. In Matlab *Upazila* (subdistrict), ICDDR,B has been maintaining a Health and Demographic Surveillance System (HDSS) since 1966 [28]. Matlab is a rural area located about 55 km southeast of Dhaka. The Health and Demographic Surveillance site Abhoynagar is located in the south-western part with a total surveillance population 34,717 and Mirsarai, with a surveillance population 39,025, is located in the south-eastern part of Bangladesh [29]. The HDSS provided the sampling frame for our study.

Data for this study was taken from the NCD risk factors survey conducted in Bangladesh in 2005 as part of the 9 HDSS areas in 5 Asian countries using the WHO STEPS methodology. The detail method has been described elsewhere [30-32]. Briefly, the STEPS approach is composed of 3 steps: structured questionnaire, physical measurements, followed by biochemical analysis of blood samples.

In a representative sample of adults aged 25 to 64 years, first two phases of STEPS were implemented in each surveillance sites. Total sample size calculated is 2000 X 3. Considering the possibility of large number of males being absent at the daytime, a sample of 2800 X 3 males and females was drawn from this population. In each 10-year range age group there were 350 samples per site with equal number of males and females. All consenting adults were interviewed. Absentees were approached three times, when unsuccessful, were excluded from the study. The participation rate in this study is 72.55%.

#### Data collection procedure

Questionnaires were translated into Bangla from English and then back translated to English identify the consistencies. Questionnaires were pretested in the field in a pilot phase, before the actual data collection. Six skilled field workers per site were recruited for the study and re-trained on standard methods of obtaining physical measurements. To ensure the quality of data, independent field worker checked 3% of data and held periodic meetings to provide necessary feedback to the field workers.

### Measurements

Blood pressure was measured using digital blood pressure measurement devices (Omron M3). Blood pressure was measured and recorded during household visits following the STEPS methods (at the right arm at heart level after a period of 5 minutes of rest). The averages of the last two measurements, from the three readings, were used in the analysis.

Participants were with lightweight wears and barefooted during measuring their weight and height. Electronic scales (Seca Gmbh, Hamburg, Germany) were placed at flat surface to measure weight (to the nearest 10 grams). Height was measured using portable stadiometer (to the nearest 0.1 cm). Waist circumference was measured by putting the measuring tape at the midpoint between the lower margin of the last rib and the top of the hip bone (at the level of umbilicus) at the end of expiration. Non-elastic tape (Seca Gmbh, Hamburg, Germany) was used to measure waist circumference, to the nearest 0.1 cm.

During the household visits, questionnaires on tobacco smoking and chewing, intake of fruit and vegetable and patterns of physical activities were administered. For tobacco consumption, information on current and ever smoking along with use of smokeless tobacco (tobacco leaf, *goul*, *noshi*, and *zarda*) were collected. Participants were asked about their fruits and vegetables consumption in a typical week, number of days and the number of servings on those days was collected. We have analysed fruits and vegetable consumption as number of times per week.

The Global Physical Activity Questionnaire Version 2 was used to collect the information on physical activities [33]. We have used the asset index data as substitute for socio-economic status, collected separately from the surveillance database of HDSS.

#### Definitions

Pre-hypertension: Pre-hypertension was defined as a systolic blood pressures between 120 to 139 mmHg and/or a diastolic blood pressures between 80 and 89 mmHg according to the 7th report of JNC [10].

Hypertension: Hypertension was defined if the systolic blood pressure was ≥140 mm Hg and/ or the diastolic blood pressure was ≥90 mmHg, or if the participants were taking antihypertensive medicine [10].

Asset index: Detail calculation of the asset index is available elsewhere [34]. Briefly, household assets and housing characteristics, such as beddings, kitchenware & utensils, furniture & cupboards, radio, television, bicycle, boat, cows and electricity were considered in calculating asset index. A single variable was produced combining these asset characteristics, and ranked in ascending order. The poverty quintiles were then developed by dividing this variable into 5 equal groups.

Body mass index (BMI): BMI is calculated using the formula, the weight in kilograms divided by the square of the height in meters (kg/m2). BMI was categorized into four groups as <18.5, 18.5-22.9, 23.0-27.4 and ≥27.5 kg/m^2^.

#### Statistical analysis plan

Descriptive analysis was performed for the socio-demographic and other predictive factors. For continuous and categorical variables, mean (standard deviation, SD) and proportion were calculated, respectively. The prevalence of prehypertension and hypertension were calculated. The age of the sample population was categorized into five groups (25–39, 40–49, 50–59 and 60+ years). C*hi*-square statistics was used to compare categorical variables. Adjusted and unadjusted logistic regression analyses were performed to evaluate the association of prehypertension and hypertension with various factors. Odds ratios (OR) and 95% confidence intervals (CI) were calculated, *p* < 0.05 was used as the level of significance. In the multivariate analysis we have adjusted for the covariates age, sex, education, asset index, smoking, tobacco chewing, fruit intake, vegetable consumption, physical activities and BMI. SAS (Version 8) Statistical software was used for the analysis.

## Results

Table 1 shows the prevalence and distribution of pre-hypertension and hypertension by demographic and socio-economic status among the adults of rural Bangladesh. The prevalence of pre-hypertension and hypertension are 31.9% and 16.0%, respectively. The mean age is also different among normotensive (42.1 years), pre-hypertensive (44.5 years) and hypertensive (50.1 years) groups (p < 0.0001) (Table 1).

**Table 1:** Distribution of pre-hypertension and hypertension by socio-economic and lifestyle factors among the adults of Rural Bangladesh* (n = 6094)

**Characteristics**	**Normotension <120/80 mmHg N = 3,176**	**Pre-hypertension 120-139 or 80-89 mmHg N = 1,946**	**Hypertension ≥140 or ≥ 90 mmHg N = 972**	**p-value**
Prevalence	52.1	31.9	16	
Age, 25-39	44.0	37.3	16.9	
40-49	28.3	24.8	25.5	
50-59	20.6	25.3	35.6	
60 and above	7.3	12.6	22.1	<0.0001
Mean (SD)	42.1(10.9)	44.5(11.6)	50.1(10.3)	<0.0001
Sex men	51.0	52.8	42.6	<0.0001
Education				
No	41.9	40.6	45.3	
Primary	28.2	26.6	26.2	
Secondary	22.2	23.6	20.1	
Higher secondary	4.0	4.4	4.1	
Higher	3.7	4.8	4.3	0.9092
Mean( SD)	3.8(4.3)	4.2(4.5)	3.8(4.4)	0.0139
Asset Index				
Poorest	16.3	12.4	13.2	
Poorer	19.8	14.6	15.7	
Middle	19.1	19.1	19.9	
Less poor	19.9	23.1	19.0	<0.0001
Least poor	25.0	30.8	32.2	
Smoking	31.3	29.5	20.1	<0.0001
Chewing	35.9	34.4	40.1	0.0092
Fruit intake, Mean (SD)	2.6(4.2)	2.6(4.2)	2.2(3.9)	0.0138
Vegetable intake, Mean (SD)	15.1(9.3)	15.6(9.1)	14.1(9.1)	0.0006
Physical activity (min per day)				
Walking (SD)	36.1(61.9)	34.4(59.5)	26.3(50.5)	<0.0001
Moderate (SD)	77.3(134.2)	79.4(132.4)	60.2(113.1)	0.0004
Heavy (SD)	23.9(87.0)	19.5(76.5)	12.2(61.2)	0.0003
BMI				
<18.5	33.3	22.1	22.7	
18.5-22.9	50.4	48.6	43.2	
23.0-27.4	14.0	22.7	25.8	
≥27.5	2.2	6.6	8.2	<0.0001
Mean ± SD	20.5(8.3)	21.8(7.9)	22.1(7.1)	<0.0001

*Results are expressed as percent, unless otherwise indicated.

**Figure 1:**
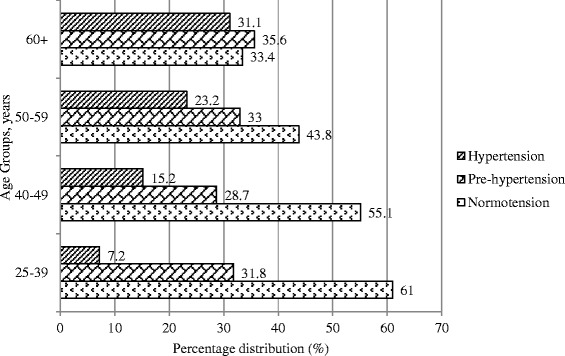
**Percentage distribution of Normotension, pre-hypertension and hypertension by age groups among the adults of rural Bangladesh.**

As with pre-hypertension, the prevalence of hypertension also increases with age (Figure 1).

**Figure 2:**
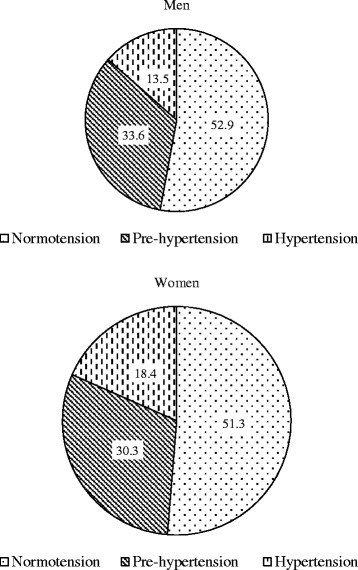
**Percentage distribution of normotension pre-hypertension and hypertension by sex among the adults of rural Bangladesh.**

The prevalence of pre-hypertension was higher in the men (33.6%) compared to the women (30.3%) (p < 0.0001). On the contrary, the prevalence of hypertension was higher among the women (18.4%) compare to the men (13.5%) (p < 0.0001) (Figure 2).

The prevalence did not vary significantly by education categories, but mean years of education are different between the pre-hypertensive (4.2 years) and the hypertensive people (3.8 years). The prevalence of pre-hypertension and hypertension increases with increasing wealth. There is a difference between the prevalence of pre-hypertension and hypertension for smoking, tobacco chewing, fruit consumption, vegetable consumption, and different levels of physical activity (Table 1).

**Figure 3:**
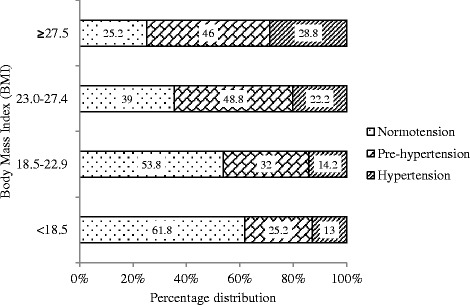
**Percentage distribution of normotension pre-hypertension and hypertension by BMI among the adults of rural Bangladesh.**

The prevalence of the pre-hypertension and hypertension is different among the BMI categories (p < 0.0001) (Table 1). The prevalence of the pre-hypertension was 48.8%, highest in the BMI category of 23.0-27.4 (overweight by the WHO standard for the Asian people), and 46% in the BMI category ≥27.5. In the lowest BMI category (<18.5) the prevalence of hypertension was 13% and the prevalence increases with increasing BMI (Figure 3).

Multivariate analysis showed that increasing age, male sex and higher BMI were positively, and fruit consumption and tobacco chewing were inversely associated with pre-hypertension (Table 2). Comparing the predictors for hypertension, multivariate analysis showed that increasing age and higher BMI (specially the overweight category) was positively associated (Table 2).

**Table 2:** **Comparison of predicting risk factors for Prehypertension (vs. Normotension) and Hypertension (vs. Prehypertension) Groups**

	**Adjusted OR (95% CI)**	
**Risk factors**	**Prehypertension vs normotensive**	**Hypertension vs pre hypertension**
Age		
25-39 yrs (reference)		
40-49	1.067(0.914 - 1.246)	2.517(1.965 -3.225)
50-59	1.517(1.286 - 1.789)	3.642(2.853 - 4.649)
60 and above	2.295(1.835 - 2.870)	4.480(3.380 - 5.939)
Sex		
Female (reference)		
Male	1.198(1.014 - 1.417)	0.927(0.732 - 1.174)
Education		
No (reference)		
Primary	0.865(0.742 - 1.008)	1.060(0.856 - 1.313)
Secondary	0.902(0.756 - 1.076)	1.092(0.846 - 1.410)
Higher secondary	0.768(0.552 - 1.068)	1.408(0.881 – 2.249)
Higher	0.936(0.670 - 1.309)	1.246(0.784 - 1.982)
Quintiles		
Poorest (reference)		
Poorer	0.958(0.769 - 1.194)	0.990(0.720 - 1.361)
Middle	1.216(0.982 - 1.506)	0.940(0.694 - 1.273)
Less poor	1.335(1.078 - 1.655)	0.691(0.508 - 0.942)
Least poor	1.248(1.000 - 1.556)	0.817(0.598 - 1.116)
BMI		
<18.5 (reference)		
18.5-22.9	1.519(1.311 - 1.760)	1.033(0.830 - 1.287)
23.0-27.4	2.459(2.028 – 2.980)	1.362(1.051 - 1.764)
≥27.5	4.667(3.346 – 6.510)	1.441(0.999 - 2.079)
Smoking		
Not smoking (reference)		
Currently smoking	0.894(0.755 - 1.057)	0.683(0.534 – 0.874)
Chewing		
Not chewing (reference)		
Currently chewing	0.86(0.75 – 0.98)	0.89(0.74 - 1.07)
Fruit consumption	0.98(0.97 – 1.00)	0.99(0.97 – 1.01)
Vegetable consumption	1.00(0.99 – 1.01)	0.99(0.98 – 1.00)
Physical activity		
Walking	1.00(1.00 - 1.00)	0.999(0.997 - 1.001)
Moderate	1.00(1.00 - 1.00)	0.999(0.998 - 1.000)
Heavy	1.00(1.00 - 1.00)	0.999(0.998 - 1.001)

Significant linear relationships of prehypertension was found with age [OR 1.019 (1.014-1.024)] (p < 0.0001) and BMI [OR1.027 (1.015-1.039) ( p < 0.0001). Linear regression for hypertension shows significant association with age [OR1.046 (1.038-1.054) (p < 0.0001) but not with BMI [OR 1.004 (0.995-1.014) (p = 0.3783).

## Discussion

This is the first study in Bangladesh reporting the prevalence and predicting factors of pre-hypertension among the rural people of Bangladesh. This study also reports the prevalence and associated factors related to hypertension. Increasing age, male sex and higher BMI are positively associated with pre-hypertension. Studying the predictors for hypertension compared with pre-hypertension, multivariate analysis showed that increasing age and higher BMI (specially the overweight category) are positively associated. The prevalence of pre-hypertension was significantly higher among the men (33.6%) compared to women (30.3%) in this population. This finding is consistent with other findings [35,36]. The prevalence of hypertension was higher in women compared to men, consistent with other findings [37]. Therefore, opportunity may exist to target men and thus protect them from developing frank hypertension. We observed a low prevalence of prehypertension but high prevalence of hypertension among female in this population, this deserves further exploration, if hormonal or other factors make women more susceptible to get hypertension quickly before going through pre-hypertension phase, and thus making women more vulnerable to hypertension related target organ damage. This also indicates that focus regarding controlling of hypertension should be in practice for this group to avoid complications from hypertension.

In this study, we have found that increasing age is an independent risk factor for pre-hypertension and hypertension. Other studies also found that age is a significant risk factor for pre-hypertension [24]. The observed association between age and hypertension is well reported [35,38]. Age is an un-modifiable risk factor; therefore other modifiable risk factors should be controlled through some interventions. For example, controlling weight may counter the age effect and delay the progression to hypertension.

Increasing BMI is found to be an independent and important risk factor for both pre-hypertension and hypertension in this study. Relationship of higher BMI with pre-hypertension was also observed in other studies [35,39] and the association of high BMI and hypertension is well established [38,40]. Increasing BMI, even the normal range compared to the below normal range, is associated with pre-hypertension. Evidence exist that overweight and obesity are the strongest predictor of prehypertension [41,42]. BMI is the robust predictor of pre-hypertension, odds ratio of obese category is the highest among all the significant predictors (OR 4.67, CI 3.35-6.51). In this study, only the overweight category is associated with hypertension when compared with people with pre-hypertension. There is a clear relationship between body weight with pre-hypertension and hypertension in this study population. Body weight is the balance between consumption and expenditure of energy. Further research is needed to examine the role of diet and effect of physical activity on pre-hypertension and hypertension in this population.

In our study, the better off (least poor) people are more likely to be pre-hypertensive. The relationship between poverty quintile and hypertension is similar in this study.

Cohort studies confirmed that the risk of cardiovascular mortality starts with blood pressure level as low as 115/75 mm Hg and increases in a linear approach for every 20/10 mm Hg rise of blood pressure [8]. Recognizing and classifying individuals with pre-hypertension directs us to concentrate on individuals with increased CVD risk and in whom valuable therapeutic interventions are to apply to prevent or delay the onset of hypertension [43].

Prehypertension is now recognized as an important public health problem. Nevertheless, the prevalence of prehypertension varies considerably in different countries, which may be due to ethnicity, as well as various local factors, such as climate and lifestyle. Less is known about the factors associated with prehypertension among the Asian people, let alone Bangladeshis. Prehypertension increases the risk of cardiovascular diseases, and many a times clustered with other risk factors, such as BMI. It is now known that Asians developed CVD at a lower level of BMI compared to the Caucasians.

Our findings may not be generalized for the whole country, as data comes from the HDSS, which provide mainly surveillance system in rural settings. Nonetheless, 75% of the population lives in rural area in Bangladesh [44]. We have used the self-reported information on tobacco use, physical activity, fruit & vegetable consumption, which might have compromised the validity.

There were several strengths in this study. The rural surveillance sites cover a wide geographical area and scattered throughout the country. The accuracy of our finding is also supported by our large sample size. Usually the surveillance population is well informed and accustomed about the timely data collection and a high response rate is generally observed. The data comes from the HDSS, which helped us to measure the prehypertension burden among the surveillance population, whereby researchers may follow this group to predict future patterns of hypertensive diseases, along with stroke and other cardiovascular diseases; where it will be easy to monitor being under the surveillance system.

## Conclusion

Approximately one third of the adult population of rural Bangladesh is affected with pre-hypertension and one sixth is affected with hypertension. This places a great challenge ahead. Serious evaluation of this at-risk group is highly warranted. People with pre-hypertension can serve as a group to guide valuable interventions to control and prevent cardiovascular diseases.

## Abbreviations

WHO STEPS: World Health Organization STEPwise approach to surveillance
INDEPTH: International Network for the Demographic Evaluation of Populations and Their Health in Developing Countries
NCD: Non Communicable Diseases
ICDDR,B: International Centre for Diarrhoeal Diseases Research, Bangladesh
HDSS: Health and Demographic Surveillance System
CVD: Cardio Vascular Diseases
SBP: Systolic Blood Pressure
DBP: Diastolic Blood pressure
JNC7: The 7th report of the Joint National Committee on Prevention, Detection, Evaluation, and Treatment of High Blood Pressure
SD: Standard Deviation
OR: Odds Ratio
CI: Confidence Interval
BMI: Body Mass Index
